# Food insecurity and associated factors among HIV-infected individuals receiving highly active antiretroviral therapy in Jimma zone Southwest Ethiopia

**DOI:** 10.1186/1475-2891-11-51

**Published:** 2012-07-23

**Authors:** Ayele Tiyou, Tefera Belachew, Fisehaye Alemseged, Sibhatu Biadgilign

**Affiliations:** 1 Jimma University, Public Health Faculty, P.O.Box 170998, Addis Ababa, Ethiopia; 2 Professor of Human Nutrition, Head Research and Publication Office, Jimma University, P.O.Box: 1104, Jimma, Ethiopia; 3 Assistant professor of Epidemiology, Department of Epidemiology, Jimma University, P.O.Box: 1104, Jimma, Ethiopia; 4 Department of Epidemiology and Biostatistics, Jimma University, College of Public Health and Medical Science, P.O.Box 24414, Addis Ababa, Ethiopia

**Keywords:** Food insecurity, HAART, PLWHA, HIV/AIDS, Ethiopia

## Abstract

**Background:**

In resource limited settings, many People Living with HIV/AIDS (PLWHA) lack access to sufficient quantities of nutritious foods, which poses additional challenges to the success of antiretroviral therapy (ART). Maintaining adequate food consumption and nutrient intake levels and meeting the special nutritional needs to cope up with the disease and the ART are critical for PLWHA to achieve the full benefit of such a treatment.

**Objective:**

To determine the prevalence and correlates of food insecurity among HIV-infected individuals receiving highly active antiretroviral therapy in resource-limited settings.

**Methods:**

A cross sectional study was carried out from January 1, 2009 to March 3, 2009 at ART clinic at Jimma University specialized hospital (JUSH) in Ethiopia. We used multivariable logistic regression model to compare independent risk factors by food insecurity status among 319 adult PLWHA (≥18 years) attending ART Clinic.

**Results:**

A total of 319 adult PLWHA participated in the study giving a response rate of 100%. Out of 319 PLWHA the largest numbers of participants, 46.4% were in the age group of 25-34 years. The overall 201(63.0%) PLWHA were food insecure. Educational status of elementary or lower [OR = 3.10 (95%CI; (1.68-5.71)], average family monthly income <100 USD [OR = 13.1 (95% CI; (4.29-40.0)] and lower food diversity [OR = 2.18 (95%CI; (1.21-3.99)] were significantly and independently associated with food insecurity.

**Conclusion:**

Food insecurity is a significant problem among PLWHA on HAART. Lower educational status and low family income were the predictors of food insecurity. Food security interventions should be an integral component of HIV/AIDS care and support programs. Special attention need to be given to patients who have lower educational status and are members of households with low income.

## Introduction

The estimated number of persons living with Human Immunodeficiency Virus (HIV) worldwide in 2009 was 33.3 million. From these figures in 2009 the number of adult People Living with HIV/AIDS (PLWHA) was 30.8 million [1]. Food insecurity and poor nutritional status may hasten progression to Acquired Immuno Deficiency Syndrome (AIDS)-related illnesses, undermine adherence and response to antiretroviral therapy, and exacerbate socioeconomic impacts of the virus. HIV infection itself weakens food security and compromises nutritional status by reducing work capacity and productivity, and jeopardizing household livelihoods [2].

Food insecurity has been defined as the ‘limited or uncertain availability of nutritionally adequate and safe foods, or limited or uncertain ability to acquire acceptable foods in socially acceptable ways’ [3]. According to World Bank, food security is defined as “access by all people at all times to enough food for an active, healthy life” [4]. The concept of food insecurity includes problems with the quantity and quality of the food available, uncertainty about the supply of food, and experiences of going hungry [5].

In resource limited settings, many PLWHA lack access to sufficient quantities of nutritious foods, which poses additional challenges to the success of Anti Retroviral Therapy (ART) [2,6]. There is a risk that declining food security will lead some people to discontinue treatment, due to a lack of adequate food (which is necessary for taking antiretroviral drugs)[6].The threat that HIV/AIDS poses on food security was first recognized in the late 1980s and early 1990s [7-9]. Maintaining adequate food consumption and nutrient intake levels and meeting the special nutritional needs to cope up with the disease and the ART are critical for PLWHA to achieve the full benefit of such a treatment [10]. Nutritional support can be taken as an important factor for ART adherence as lack of food was one of the causes of non-adherence in antiretroviral treatment program [11].

The combined impacts of food insecurity and HIV/AIDS place further strain already limited household resources as affected family members struggle to meet household food needs [12]. However; little is known about the food insecurity and associated factors among adults PLWHA on ART in the Ethiopia context. The objective of this study was to determine the proportion and correlates of food insecurity among HIV-infected individuals receiving highly active antiretroviral therapy in resource-limited settings in Jimma University Specialized hospital in Southwest of Ethiopia.

## Methods

### Study setting and context

A cross-sectional facility based study was conducted in Jimma University Specialized Hospital which gives service to more than 10,000,000 people living in south western part of Ethiopia. The Antiretroviral (ART) clinic of the hospital started to provide service to People Living with HIV/AIDS (PLWHA) in 2002. Since its establishment, the clinic had 3,700 individuals following care and treatment; at the time of the study 1,190 were started with ART. This study was conducted from January 1, 2009 to March 3, 2009.

### Participants

The source populations were all PLWHA on Highly Active Antiretroviral Treatment (HAART) registered and have a follow up for their treatment in Jimma University Specialized Hospital (JUSH) at the time of the study. The study population were adults who fulfilled the inclusion criteria- all PLWHA on HAART whose age is >18 years regardless of their treatment regimen, duration of follow up category during the study period, and available during data collection period. Those patients on HAART whose age is <18 years, adults (>18 years) PLWHA who had been on treatment for less than 3 months period; and participants reported diabetes mellitus, current pregnancy (since they need additional food/nutrients than the others) at screening were excluded from the study.

The sample size was calculated using Epi-info version 6.04 StatCalc taking the proportion of respondent considered as food insecure to be 50%, a precision of 5% and with 95% confidence level. After adding 10% for non-response, an overall sample size of 319 was obtained. The study participants were selected randomly using a computer generated simple random based on patient ART unique identification number. Random selection of participants was done prior to patients presenting to clinic.

### Data collection procedures

Data on variables including food security, food aids for PLWHA, age, sex, associated diseases were collected using an interviewer administered pretested Amharic version questionnaire. The questionnaire was adapted from other studies [13,14], translated into Amharic and back translated to English by other person to check for its consistency. Food security scales which capture several dimensions of food security including worry over the adequacy of the food supply, concern about the quality of the food supply, changes in eating patterns in response to food shortages and the availability of resource based on the three pillars of food security measurement scales; availability, accessibility and utilization was used [15-17].

### Measurements

#### Food security and dietary diversity scales

There were a total of 31 questions measure “food security and dietary diversity” while only 10 questions specifically measure food security of PLWHA in the last 4 weeks before the survey. The responses were coded in such a way that affirmative answer is given a score of 1 and non affirmative answer is given a score of 0. When a PLWHA has ≤ 2 affirmative answers, out of the 10 questions were considered as food secure; while those with more than 2 affirmative answers were considered as food insecure. Seven of the questions which indicate the number of eating occasion and twelve of the questions were used to assess dietary diversity. Individuals who scored more than the mean value were considered as having a good diversity scores [18]. The way the dietary diversity was calculated should be made clear as follows: The dietary diversity was assessed using a the questions specifically covered food consumption during the past 24 hr period containing Cereals, Miscellaneous, Oils/Fats, Honey/Sugar, Legumes, Root/Tubers, Fruits ,Vegetables, Meat, Milk/Milk products, Eggs, Fish/Sea foods that are commonly consumed in the study area. Participants were asked to report the frequency of consumption of each food using the past 24 hours. Participants received 1 point if they consumed at least once during the last 24 hours of the foods within each subgroup and 0 points if they never consumed the food. The food items were grouped into six according to the food guide pyramid (US Department of Agriculture, 1992). A Dietary Diversity Score (DDS) was calculated as the sum of the food groups consumed over 24 hours. The dietary diversity score ranged from one to twelve. The mean (±SD) dietary diversity score in the study group was 6.29 (± 1.9). Then tertiles of the dietary diversity score were computed with the highest tertile defined as diversified diet, while the lowest two tertiles combined were labeled as undiversified diet. Cronbach’s Alpha values for the scale used in this study showed 0.92 for food security and 0.73 for meal frequency and 0.75 for dietary diversity score.

### Statistical analysis

The data were edited, coded, entered into a computer, and analyzed using SPSS for windows version 16.0. Descriptive statistics was done to assess basic client characteristics and proportion of food insecurity. Bivariate analysis was done to determine statistical association between explanatory variables and food insecurity. All variables that were associated with food insecurity in bivariate analyses were entered into multivariable forward stepwise elimination logistic regression model. P-values at the level of significance of 5% were considered statistically significant. The reliability of items of the scale was evaluated using Cronbach’s alpha. A cut-off value of 0.7 and above was used as acceptable internal consistency level [19]. Intensive two day training was given to all supervisors and data collectors before the process of data collection. To assure quality of the data, the questionnaire was pre-tested on 21 PLWHAs (5% of the sample size) and modifications were incorporated into the questionnaire.

### Ethical consideration

Ethical clearance was obtained from Institutional Ethical Review Committee of Jimma University and official letter of co-operation was given to Jimma University Specialized Hospital. All study participants gave a verbal informed consent for participating in the study.

## Results

A total of 319 adult PLWHA participated in the study giving a response rate of 100%. Out of 319 PLWHA the largest numbers of participants, 46.4% were in the age group of 25-34 years with the mean (±SD) age of the respondents was 35.08(±7.73) years. Larger proportion of the respondents were from Jimma City (85.0%), Oromo by ethnicity (44.5%), individual and family monthly income of the participants was 30 and 35 USD respectively, Orthodox Christians by religion (50.8%), married (48.6%) and had attended elementary school(50.8%). Two hundred forty four (76.5%) were employed by occupation, 54.5% were lived with their parents, and 78.7% didn’t have any food aids from any organization (Table 1).

**Table 1:** Socio-demographic and Economic characteristics of the study participants, JUSH, Southwest Ethiopia, 2009

**Variables**	**Food insecurity status**		**Total frequency**	**P-value**
	**Food secure**	**Food in-secure**		
Sex				
Male	58 (40.3)	86 (59.7)	144 (45.1)	0.270
Female	60 (34.3)	115 (65.7)	175 (54.9)	
Age				
18-24	6 (40.0)	9 (60.0)	15 (4.7)	0.261
25-34	56 (32.7)	115 (67.3)	148 (46.4)	
35-44	36 (39.1)	56 (60.9)	115 (36.1)	
≥45	20 (48.8)	21 (51.2)	41 (12.9)	
Permanent address				
Jimma	82(30.3)	189 (69.7)	271 (85.0)	<0.001
Out of Jimma	36(75.0)	12 (25.0)	48 (15.0)	
Marital Status				
Married	60 (38.7)	95 (61.3)	155 (48.6)	0.096
Single	31 (46.3)	36 (53.7)	67 (21.0)	
Windowed	13 (30.2)	30 (69.8)	43 (13.5)	
Divorced /Separated	14 (25.9)	40 (74.1)	54 (16.9)	
Religion				
Orthodox Christians	63 (38.9)	99 ( 61.1)	162 (50.8)	0.360
Muslim	29 (37.2)	49 (62.8)	78 (24.5)	
Protestant	20 (29.4)	48 (70.6)	68 (21.3)	
Others*	6 (54.5)	5 (45.5)	11 (3.4)	
Ethnicity (N = 319)				
Oromo	61 ( 43.0)	81 (47)	142 (44.5)	0.062
Amhara	25 (29.8)	59 (70.2)	84 (26.3)	
Dawro	11 (27.5)	29 (72.5)	40 (12.5)	
Kefa	9 (37.5)	15 (62.5)	24 (7.5)	
Gurage	3 (20.0)	12 (80.0)	15 (4.7)	
Others**	9 (64.3)	5 (35.7)	14 (4.4)	
Average family monthly income (USD)***				
<100	62 (24.9)	187 (75.1)	249 (81.4)	<0.001
≥100	51 (36.9)	6 (10.5)	57 (18.6)	
Occupation				
Employed@	93 (78.8%)	151 (75.1%)	244 (76.5%)	0.453
Unemployed	25 ( 21.2%)	50 (24.9%)	75 (23.5%)	
Living With				
Alone	23 (29.9)	54 (70.1)	77 (24.1)	0.365
Family	24 (44.4)	30 (55.6)	54 (16.9)	
Parents	65 (37.4)	109 (62.6)	174 (54.5)	
Other				
Educational Status	6 (42.9)	8 (57.1)	14 (4.4)	<0.001
Elementary or less	39 (20.1)	155 (79.9)	194 (60.8)	
Secondary and above	79 (63.2)	46 (36.8)	125 (39.2)	
Average family monthly income (USD)				
<100	62 (24.9)	187 (75.1)	249 (81.4)	<0.001
≥100	51 (36.9)	6 (10.5)	57 (18.6)	
Average individual monthly income (USD)				
<100	64 (28.3%)	162 (71.7%)	226 (84.6)	<0.001
≥100	37 (90.2%)	4 (9.8%)	41 (15.4)	
Food Diversity				
High	72 (49.6%)	76 (50.4%)	148 (46.4)	<0.001
Low	46 (27.0%)	125 (73.0%)	171 (53.6)	
Do you get money support from any organization?				
Yes	11 (20.8%)	42 (79.2%)	53 (16.6)	0.009
No	107 (40.2%)	159 (59.8%)	266 (83.4)	
Do you get any food ration from any organization?				
Yes	12 (17.9%)	56 (82.4%)	68 (21.3)	<0.001
No	106 (42.2%)	145 (57.8%)	251 (78.7)	
Body mass index (BMI)				
<18.5	19 (30.2)	44 (69.8)	63 (19.7)	0.210
>=18.5	99 (38.7)	157 (61.3)	256 (80.3)	
Food Aids				
Present	12 (17.6)	56 (82.4)	68 (21.3)	<0.001
Absent	106 (42.2)	145 (57.8)	251 (78.7)	

**Catholic, Jova witness, Farmer, Bar.

*Tigre, Yem, Wolayita, Kenbata, Sidama, Bench.

***Exchange rate 1 USD = 10.0 Ethiopian Birr (ETB).

@ Government, Employee of private organization/enterprise and NGO employee.

Food insecurity and dietary diversity were significantly correlated at P = 0.001. Overall, 63.0% PLWHA were food insecure (Cronbach’s alpha = 0.92) and 59.6% had a meal frequency less than the mean value (Cronbach’s alpha = 0.73) and 55.8% ate less than the mean food diversity score (Cronbach’s alpha = 0.75) in the past 24 hours. Over half of the respondents (51.1%) ate 3 times in a day and 12.5% ate more than 4 times in the day in the past 24 hour period. Hundred and forty one subjects (44.2%) stated that they took more than the mean food category types and from those food items almost all of the respondents (98.1%) ate cereals, 240(89.3%) oils/fats, 151(47.3%) fruits and (62.7%) vegetables food group and (15.4%) of the participants consumed less than 5 food groups in the past 24 hour period.

After controlling the effects of other covariates, variables which were significantly associated on the bivariate analysis with food insecurity (educational status, average family monthly income (USD), average individual monthly income (USD), food diversity, getting money support and food ration from any organization) were fitted into multivariable logistic regression model; three variables were found to be predictors of food insecurity status of PLWHA. Individuals with educational level of elementary or lesser were 3.10 times more likely to be food insecure than those who had higher educational status [OR = 3.10 (95%CI; (1.68-5.71)]. Average family monthly income was the other predictor of food insecurity; families earning less than 100 USD were 13.1 times more likely to be food insecure [OR = 13.1 (95% CI; (4.29-40.0)] and individuals with low food diversity were 2.18 times more likely to be food insecure than those who had high food diversity [OR = 2.18 (95%CI; (1.21--3.99)] (Table 2).

**Table 2:** Association of variables with food insecurity among PLWHA on HAART, Jimma University Specialized Hospital, 2009

**Variables**	**Food insecurity status**		**Crude OR(95% CI)**	**P-value**	**Adjusted OR(95% CI)**	**P-value**
	**Food secure**	**Food in-secure**				
Educational Status				<0.001		<0.001
Elementary or less	39 (20.1%)	155 (79.9%)	6.82 (4.12-11.31)		3.10 (1.68-5.71)	
Secondary and above	79 (63.2%)	46 (36.8%)	1.00		1.00	
Average family monthly income (USD)				<0.001		<0.001
<100	62 (24.9%)	187 (75.1%)	25.6 (10.5-62.6)		13.1 (4.29-40.0)	
≥100	51 (36.9%)	6 (10.5%)	1.00		1.00	
Food Diversity				<0.001		0.01
Low	72 (49.6%)	76 (50.4%)	2.67 (1.67-4.26)		2.18 (1.21--3.99)	
High	46 (27.0%)	125 (73.0%)	1.00		1.00	
Do you get money support from any organization?				0.009		
Yes	11 (20.8%)	42 (79.2%)	2.57 (1.27-5.21)			
No	107 (40.2%)	159 (59.8%)	1.00			
Do you get any food ration from any organization?				<0.001		
Yes	12 (17.9%)	56 (82.4%)	3.4 1(1.74-6.68)			
No	106 (42.2%)	145 (57.8%)	1.00			

Adjusted for age, residence, occupation, financial support, food ration.

Regression method: forward stepwise elimination logistic regression model.

After controlling the effects of other covariates, variables which were significantly associated on the bivariate analysis with dietary diversity (educational status, average family monthly income (USD), average individual monthly income (USD), permanent residence ,occupational status , body mass index (BMI), food insecurity ,meal frequency) were fitted into multivariable logistic regression model: two variables were found to be predictors of dietary diversity status of PLWHA. Individuals whose educational status of elementary or less is 51% less likely to diversified their food/meal than those who had higher educational status counterpart [OR = 0.49 (95% CI: (0.30-0.80)]. Individuals with lower meal frequency were 57% less likely [OR = 0.43 (95% CI: (0.26-0.71)] to diversified their food/meal than high meal frequency individuals (Table 3). Regression diagnostic procedures was carried out and showed no evidence of multicollinearity and substantial influence from outliers.

**Table 3:** Association of variables with dietary diversity among PLWHA on HAART, Jimma University Specialized Hospital, 2009

**Variables**	**Dietary diversity**		**Crude OR(95% CI)**	**P-value**	**Adjusted OR(95% CI)**	**P-value**
	**Low**	**High**				
Educational Status				<0.001		0.005
Elementary or less	126 (64.9%	68 (35.1%)	0.38 (0.24-0.61)		0.49 (0.30-0 .80)	
Secondary and above	52 (41.6%)	73 (58.4%)	1.00		1.00	
Meal frequency				<0.001		0.001
Low	125 (65.8%)	65 (34.2%)	0.36 (0.23-0.57)		0.43 (0.26-0 .71)	
High	53 (41.1%)	76 (58.9%)	1.00		1.00	
Food Insecurity				<0.001		
Food Secured	48 (40.7%)	70 (59.3%)	2.67 (1.67-4.26)			
Food Insecured	130 (64.7%)	71 (35.3%)	1.00			
Average family monthly income (USD)				0.001		
<100	152 (61.0%)	97 (39.0%)	0.37 (0.21-0.67)			
≥100	21 (36.8%)	36 (63.2%)	1.00			
Permanent residence				0.034		
Jimma	158 (58.3%)	113 (41.7%)	1.00			
Outside Jimma	20 (41.7%)	28 (58.3%)	1.96 (1.05-3.65)			
Body mass index (BMI)				0.028		
<18.5	43 (68.3%)	20 (31.7%)	0.52 (0.29-0.93)			
>=18.5	135 (52.7%)	121 (47.3%)	1.00			

Adjusted for age, residence, income, BMI, food insecurity.

Regression method: forward stepwise elimination logistic regression model.

## Discussion

This study focused on determining the proportion, factors affecting of food insecurity among PLWHA taking antiretroviral therapy. Majority 201 (63.0%) of the study participants were food insecure which is higher than the reports form study done in British Columbia, Canada [20] where 48% of PLWHA were food insecure while it is lower than the one reported from Dire Dawa (Ethiopia) [21], 72.4% of the households were food insecure. This might be due to the variation in the socio economic status of the two study areas and the measurement taken in the food security status at household level in case of Dire Dawa while the current study assessed the individual food insecurity experiences of PLWHA.

The proxy indicators of food security status used in this study to support the ten items for assessment of food security also showed that significant number of PLWHA on ART 59.6% and 55.8% had low mean meal frequency and dietary diversity score, respectively. Similar findings were documented in Côte d’Ivoire and Uganda [22,23].

Educational status was one of the predictors of food insecurity with PLWHA’s whose educational status is lesser than elementary level being more likely to be food insecure than those who educated higher than elementary level. This finding is consistent with the findings of other reports [21,24,25]. Educated PLWHA will have an opportunity to involve in better income generation activity than less educated and may fulfill their dietary needs. Average family monthly income was one also of the predictors of food insecurity status with PLWHA in households earning average family monthly income less than 100 USD per month being more likely to be food insecure compared to those earning more. Another study also reported that PLWHAs earning less than 10, 000 Canadian dollars per year were 4 times more likely to be food insecure [21]. Findings of the longitudinal study done in Jimma zone to assess adolescent’s food security also showed family socioeconomic status has significance association with food insecurity [26]. People living in food constrained households share similar shocks which might be rather worse for PLWHAs owing to the discrimination that becomes fueled due to the shortages of food or others resources to purchase food. This has significant programmatic implication in that addressing the poverty and food insecurity issues among PLWHA is a critical element in achieving a better treatment and clinical outcome to ART. PLWHAs might even face economic problems due to cut down of their earnings due to frequent sickness days that they have passed. Additionally, unemployment promotes poverty, which contributes to food insecurity [25,27,28]. In the same manner, the mood changes produced by the lack of a steady job can impact compliance with treatment and medical appointments related to the person’s HIV condition [29]. The lessons learned from the early phase of the HIV Equity Initiative (HEI) was that the context of poverty factors such as lack of access to transport, food insecurity, and user fees for medical care, posed more significant barriers to adhering to long-term therapy than a patient’s individual behavior [30] and the most likely cause of non adherence to antiretroviral (ARV) drug therapy [31].

The other pertinent finding is the low food diversity and meal frequency which are directly related with food insecurity. Our study showed that those PLWHA who had lower than the mean diversity score were 2.18 times more likely to be food insecure which is consistent with the finding reported in another study [32]. The majority of the study households reported to have consumed fewer than six food groups, which was mainly cereals. This is evidence in a study conducted in Uganda which showed a significant increase in the number of food groups consumed with increased food security score [23].

The findings of this study should be interpreted with some limitations. Because it was conducted at a single site, the findings may not be generalizable to dissimilar clinical settings and a result of cross-sectional study design nature, the temporal sequence of events cannot be determined. Recall bias and social desirability bias are also potential limitations that may have been encountered in this study.

## Conclusions

In conclusion, food insecurity was high among HAART treated people living with HIV/AIDS in Jimma zone South west Ethiopia. The proxy indicators used to support food security like food diversity and meal frequency also showed a significant numbers of PLWHA took less than the mean eating occasions and food diversity which is comparable to similar studies done in developing countries. Predictors of food insecurity were having lower education status, low food diversity and average family monthly income less than 100 USD. This calls for integration of ART program with food security interventions at health facilities level. Social support and income generation strategies are recommended to mitigate further vulnerability PLWHA of in coping with food insecurity. Further studies with different study design are needed to asses the food insecurity situations.

## Competing interests

The authors’ declare that they have no competing interests.

## Authors' contributions

AT conceived and designed the study, performed analysis and interpretation of data and drafted the manuscript, TB, FA and SB assisted with the design, interpretation of data and the critical review of the manuscript. All authors approved and read the final manuscript. All authors participated in critical appraisal and revision of the manuscript.
